# G Protein-Coupled Receptor Kinase 2 as Novel Therapeutic Target in Fibrotic Diseases

**DOI:** 10.3389/fimmu.2021.822345

**Published:** 2022-01-17

**Authors:** Nan Li, Shan Shan, Xiu-Qin Li, Ting-Ting Chen, Meng Qi, Sheng-Nan Zhang, Zi-Ying Wang, Ling-Ling Zhang, Wei Wei, Wu-Yi Sun

**Affiliations:** Institute of Clinical Pharmacology, Anhui Medical University, Key Laboratory of Anti-inflammatory and Immune Medicine, Ministry of Education, Collaborative Innovation Center of Anti-inflammatory and Immune Medicine, Hefei, China

**Keywords:** G protein-coupled receptor kinase 2 (GRK2), G protein-coupled receptors, fibrosis, phosphorylation, ECM - extracellular matrix

## Abstract

G protein-coupled receptor kinase 2 (GRK2), an important subtype of GRKs, specifically phosphorylates agonist-activated G protein-coupled receptors (GPCRs). Besides, current research confirms that it participates in multiple regulation of diverse cells *via* a non-phosphorylated pathway, including interacting with various non-receptor substrates and binding partners. Fibrosis is a common pathophysiological phenomenon in the repair process of many tissues due to various pathogenic factors such as inflammation, injury, drugs, etc. The characteristics of fibrosis are the activation of fibroblasts leading to myofibroblast proliferation and differentiation, subsequent aggerate excessive deposition of extracellular matrix (ECM). Then, a positive feedback loop is occurred between tissue stiffness caused by ECM and fibroblasts, ultimately resulting in distortion of organ architecture and function. At present, GRK2, which has been described as a multifunctional protein, regulates copious signaling pathways under pathophysiological conditions correlated with fibrotic diseases. Along with GRK2-mediated regulation, there are diverse effects on the growth and apoptosis of different cells, inflammatory response and deposition of ECM, which are essential in organ fibrosis progression. This review is to highlight the relationship between GRK2 and fibrotic diseases based on recent research. It is becoming more convincing that GRK2 could be considered as a potential therapeutic target in many fibrotic diseases.

## Introduction

G protein-coupled receptor kinase 2 (GRK2) is a ubiquitous member of G protein-coupled receptors (GPCRs) kinase family, which contains a group of seven serine/threonine protein kinases that is capable of specific recognition and phosphorylation of GPCRs (1). Apart from well-characterized mechanisms that GRKs mediate GPCRs desensitization (2), GRK2 also participates in the regulation of an enormous range of non-GPCRs substrates, even more becoming a vital integrative node in amount processes of signals transduction (3). Fibrosis is the final stage of a chronic inflammatory response, characterized by abnormal production of the extracellular matrix (ECM). Continuous progress can cause organ malfunction, and even failure, seriously threatening human health (4). Organ fibrosis is an illness progression caused by chronic inflammation and accumulation of fibrous tissue. To date, it is accounting for 45% of all-cause mortality world-wide (5). On the contrary, scientific discoveries have confirmed that early stage of fibrotic diseases is reversible, the effective measures contain removal or elimination of the causative agent. But it seems impossible to timely occurrence reversible and appropriately wound-healing, used to avoid complications (6, 7). Despite decades of research on fibrotic diseases, few effective and clinical anti-fibrotic drugs have been discovered yet (8, 9). Therefore, it is urgently to explore the pathological process and the underlying mechanism of fibrosis. Recently, anomalous GRK2 level and activity have been observed in various tissues during fibrosis pathophysiology such as hepatic fibrosis, myocardial fibrosis, pulmonary fibrosis, etc (10). In this review, we concisely summarize the advances of GRK2 in fibrosis, to better understand its mechanism and provide new potential therapeutic targets for fibrotic diseases.

## Regulation of GRK2 Activity Under Pathophysiology Conditions

**Figure 1:**
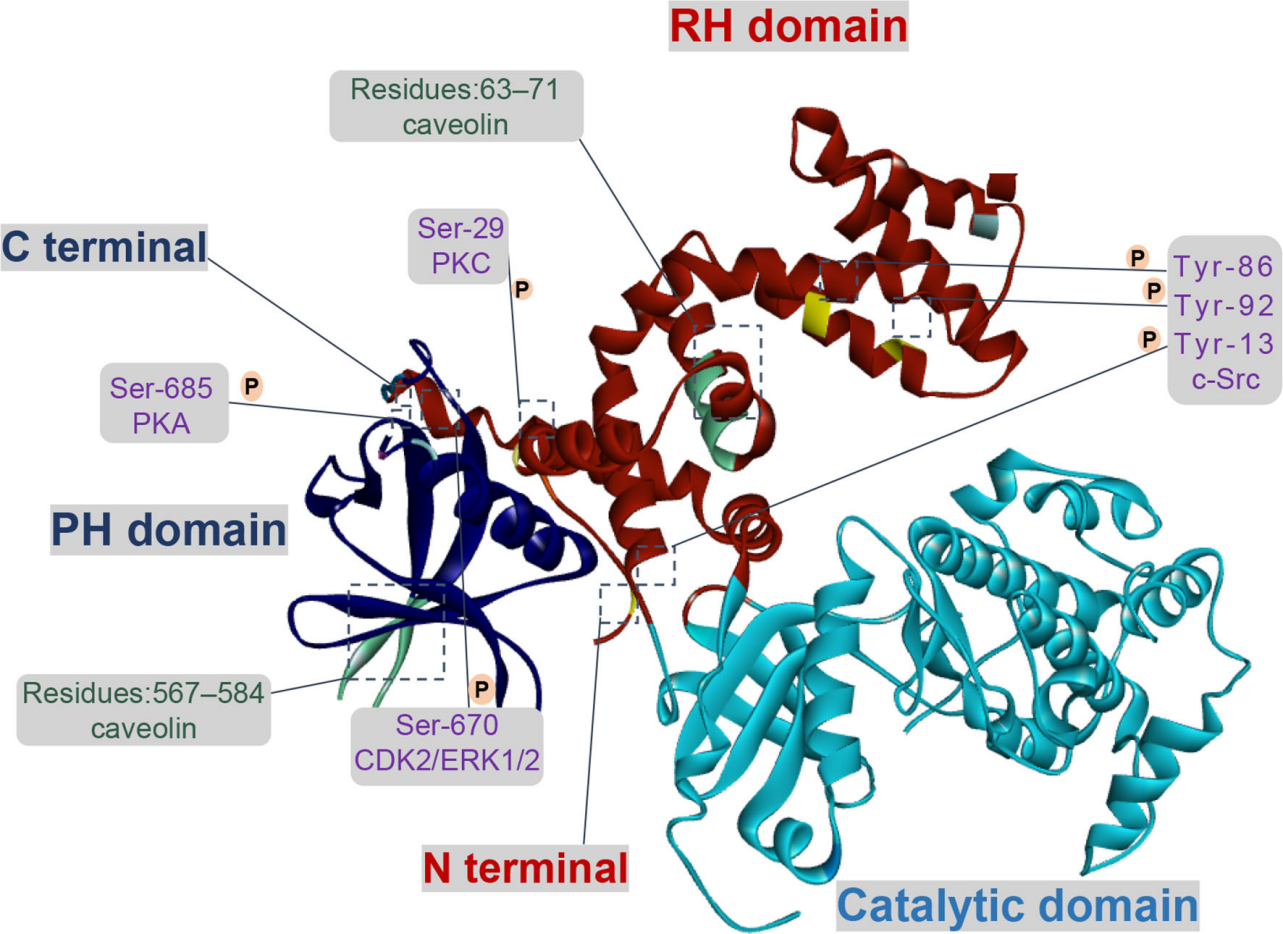
Functional domains and regulatory sites of GRK2. GRK2 contains multiple phosphorylation and non-phosphorylation sites. Phosphorylation sites of GRK2 by c-Src are Tyr-13 (N-terminal helix), Tyr-86 and Tyr-92 (RH domain). Protein kinase C (PKC) and protein kinase A (PKA) respectively phosphorylate GRK2 at Ser-29 and at Ser-685. GRK2 is phosphorylated on Ser-670 by extracellular-signal-regulated kinase 1/2 (ERK1/2) or cyclin-dependent protein kinase 2 (CDK2) down-regulates its activity. There is an interaction between caveolin and GRK2, which located in the PH domain (residues 567–584) and the N-terminal domain (residues 63–71).

Tissue fibrosis is a pathologic process, in general, injury tissues are able to restore normal organ architecture and function (11). However, if there is a dysregulation between the inflammation, the proliferative or the remodeling stages under physiological conditions, the tissue injury signals will be triggered so that contribute to fibrosis, even resulting in organ dysfunction (12). GRKs are known as a serine/threonine protein kinases family, its catalytic activity towards receptors under physiological conditions would directly determine the regulation and desensitization of GPCRs signaling pathways, as well as its interaction with additional proteins (13). At present, GRKs are comprising seven isoforms (GRK1-7) in vertebrates. More importantly, GRK2 and 3 isoforms, the second subfamily named β-adrenergic receptor kinase subfamily, are ubiquitously expressed and generally localized to the cytosol and plasma membrane (14). Furthermore, recent studies have found that there are diverse changes in the expression and activity levels of GRK2 under pathophysiology conditions. Structurally, all GRKs comprise three main modular domains: the conserved central kinase domain (KD), the N-terminal (NT) region and a C-terminal region (CT) (15). Three domains of GRK2 are placed at the vertices of a triangle, which can transduce and modulate signaling events as a single molecule. Meanwhile, this structure would be beneficial to regulate the activity of GRK2 through dynamic interactions among different GRK2 domains themselves and with different intracellular, membrane proteins (16) (**Figure 1**). Thus, further understanding of the function of GRK2 that is regulated by phosphorylation or non-phosphorylation would be contributed to improving abnormal kinase activity or expression observed in pathological disorders (17).

Currently, c-Src regulates GRK2 function through phosphorylating the N-terminal (Tyr-13) and the RH domain (Tyr-86, Tyr-92). *In vitro*, GRK2 is directly phosphorylated by c-Src and then promotes tyrosine phosphorylation of agonist-stimulated β2-adrenergic receptor (β2-AR), which relies on the recruitment of c-Src by β-arrestins (18). In HEK293 cells treated with epidermal growth factor, PDEγ, as a connected protein, plays a vital role in the interaction between c-Src and GRK2 (19). For multi-protein complex, the interaction of GRK2 with Gαq is enlarged due to tyrosine phosphorylation, suggesting a direct effect on its catalytic activity and expression in cells (20). Moreover, the activity of GRK2 is down-regulated by PKC phosphorylation at Ser-29, and the underlying mechanism which seems not to involve in inhibiting its interaction with Gβγ (21). In addition, the phosphorylation of GRK2 by PKC promotes translocation of GRK2 to cell membrane, rather than regulating its catalytic activity *in vivo*, which is conducive to the phosphorylation of GRK2 to the receptor (22).

As for the CT domain, ERK1/2 or cyclin-dependent protein kinase 2 (CDK2) can phosphorylate the Ser-670 site of GRK2, which is the Gβγ binding domain of GRK2 as well. When GRK2 is phosphorylated at Ser-670, the binding of Gβγ with GRK2 is severely interrupted, thus its catalytic activity on receptor membrane substrate is reduced (23). Moreover, insulin signaling in aorta and liver is down-regulated due to the inhibition of GRK2 activity *via* ERK1/2 phosphorylation (24). In addition, the phosphorylation of GRK2 at Ser-685 by PKA contributes to aggerating calpain-dependent GRK2 proteolysis *in vitro* or enhancing the activity of GRK2 and ultimately resulting in β2-AR phosphorylation and desensitization (25, 26). On the other hand, phosphorylation of GRK2 by PKA has no impact on its activity, but it enhances the coupling of GRK2 to Gβγ subunit and subsequent translocation to the plasma membrane, thereby promoting membrane anchoring of GRK2 and phosphorylation of receptors (27).

Effectively, the activity of GRK2 is also regulated by several additional proteins through phosphorylation and non-phosphorylation, such as clathrin, caveolin, and RKIP. Other studies revealed that upregulation of RKIP level (a negative regulator of GRK2) leads to the reduction of GRK2, thereby weakening the down-regulation activity triggered by agonists and prolonging receptor signaling (28, 29). In recent years, two caveolin-binding motifs have been reported in GRK2 at positions 567–584 and at positions 63–71, respectively located in the PH domain and in the NH2-terminal domain. When bound to caveolin-1 or caveolin-3, GRK2-mediated phosphorylation will be inhibited, presumably basal activity of GRK2 is affected by caveolin (30, 31). Currently, it is reported that GRK2 bound to clathrin depended on the motif of residues 498-502, further study demonstrates that mutation of the clathrin-binding motif of GRK2 had no obvious effect on its kinase activity and the ability to interact with β2AR (32). In addition, Zhang et al. (33) has determined that GPCRs endocytosis caused by the interaction between clathrin and GRK2 relied on the status of receptor phosphorylation.

## Relationship Between GRK2 and Fibrosis-Associated Pathways

**Figure 2:**
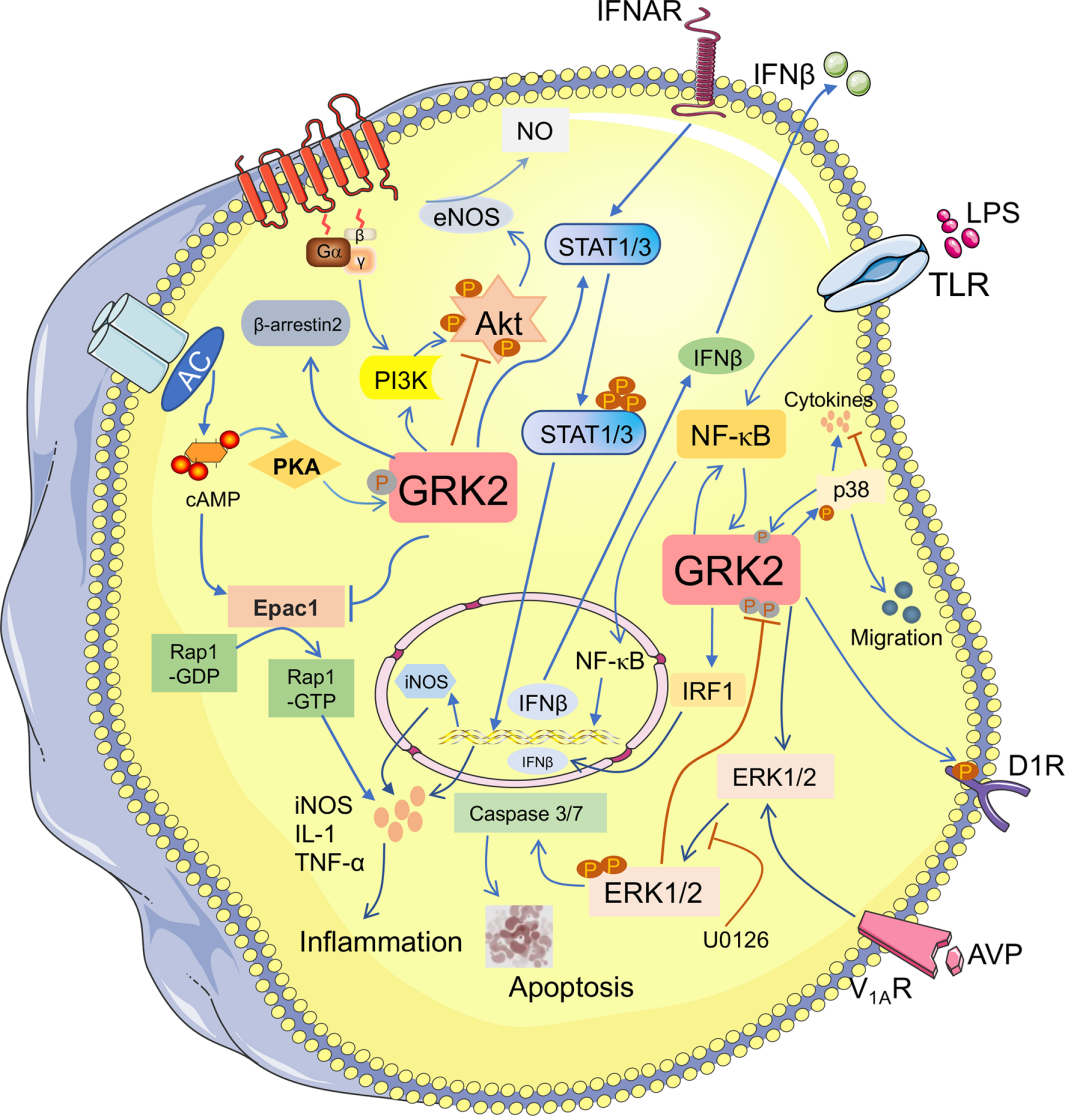
GRK2 plays a diverse role in fibrosis-associated pathways. The phosphorylation of GRK2 by different kinases (PKA) also modifies kinase activity and substrate selection. GRK2 can regulate many downstream molecules and participates in the multiple signaling pathways. It not only activates PI3K/Akt, but also inhibits Akt/eNOS pathway to lower NO production. Activation of ERK1/2 pathway contributes to apoptosis. GRK2 promotes ERK1/2 phosphorylation, while ERK1/2 inhibits GRK2 phosphorylation. GRK2 and NF-κB can advance their activation through each other. Meanwhile, GRK2 inhibits Epac1/Rap1 pathway and inhibits the release of inflammatory cytokines. However, GRK2 promotes the phosphorylation of STAT1/3, which promotes the accumulation of inflammatory cytokines and contributes to the occurrence and development of fibrotic diseases.

Continuous research indicates that GRK2-mediated cellular signal transduction is closely associated with fibrotic diseases *via* kinase-dependent and independent manners (34, 35). Most studies have demonstrated that activated GRK2 could not only regulate the GPCRs-mediated singling pathway through desensitization of GPCRs, but also regulate non-GPCRs-mediated signaling pathway (36, 37). Subsequently, tyrosine kinases and various types of receptors are investigated as downstream targets of GRK2. Furthermore, it is also engaged in regulation of protein kinases, transcription factors and their regulatory proteins (Smad2/3, IκBα) (38). Increasing evidence suggests that GRK2, which exhibits the abnormal expression or activity, participates in the regulation of fibrosis-associated pathways, thus may as an essential role engages in the development of fibrotic diseases (**Figure 2**).

### Epac1/Rap1 Signaling Pathway

Exchange protein directly activated by cAMP 1 (Epac1), as a guanine nucleotide exchange factor, is responsible for activation of the Ras superfamily, Rap1 and Rap2 (39). Thereby regulating its downstream Akt, MAPKs, PKC and other signal pathways, and then involving in multiple diseases, including fibrosis (40). Studies have revealed that the increased expression of Epac1 promoted Rap1 and fibroblast cells activation, leading to collagen accumulation (41). Niels et al. (42) strongly indicates that the low nociceptor GRK2 is conducive to Epac1/Rap1, leading to PKCϵ and MEK/ERK-dependent signaling and prolonging hyperalgesia. Studies have been shown that GRK2 directly phosphorylates Epac1 at Ser-108 depends on its kinase activity, thereby inhibiting Epac1 translocation and occurring the downregulation level of activated Rap1 (43). Based on previous studies, we have reason to speculate that GRK2 with kinase activity, as a negative regulator that regulates the Epac1/Rap1 signaling pathway, may be a therapeutic target for fibrotic diseases (44).

### AC/cAMP/PKA Pathway

It is now recognized that G-protein-adenylate cyclase (AC)-cAMP signal is one of the important transfer models in intracellular signal transduction, which plays a cascade amplification role in the transmission of external stimulus signals. cAMP, as an important second letter messenger, is produced by AC catalyze from ATP and the main function is to activate cAMP-dependent PKA. In acetaldehyde-treated primary hepatic stellate cells (HSCs), activation of AC/cAMP/PKA pathway contributes to HSCs activation and increasing the level of collagen I and III (45). Indeed, the further study investigates that the increased translocation of GRK2 by AC/cAMP/PKA, rather than phosphorylation, reducing the binding of GRK2 and ERK1/2 to inhibit ERK1/2 activation, which promotes PGE_2_-induced angiogenesis (46). At the same time, it has been found that VPAC2 receptor was phosphorylated by GRK2, accompanying with GRK2 phosphorylation at Ser-685 by PKA. Subsequently, the activated GRK2 could augment its mediated functional responses, including internalization and desensitization (47). Furthermore, GRK2 phosphorylates activated β-ARs to decouple G protein, decreasing the level of cAMP and then aggravates cardiac myocytes death. Recent study suggests inhibiting GRK2 in the cardiac fibroblasts (CF) could decrease fibrosis and fibrotic gene expression (48). These findings emphasize that a complex interaction exists among GRK2 and the AC/cAMP/PKA signaling pathway during the progression of fibrosis.

### STAT1/3 Pathway

It is now recognized that the activation of STAT is associated with the gene expression of pro‐inflammatory and pro‐fibrosis (49). Upon activation, STAT3 is involved in fibrosis through enhancing ECM production (50). Likewise, activation of JAK2-STAT3 contributes to collagen synthesis, which is associated with the formation of the liver fibrosis or in high glucose-induced CF (51, 52). GRK2 has been identified to regulate a variety of pro‐inflammatory secretions. The high expression of nitric oxide synthase (iNOS) in microglial cells is caused by GRK2, which significantly enhances the phosphorylation levels of STAT1 and STAT3 through regulating the toll like receptor (53). The upregulation of iNOS could promote the secretion of inflammatory cytokines and development of many fibrous diseases (54). In addition, the nuclear translocation of phosphorylated STAT1 and STAT3 is restrained when GRK2 siRNA is transfected, in turn blocking the expression of iNOS (55). Therefore, these observations suggest a potential effect on GRK2-regulated the STAT1/3 pathway in various aspects of fibrosis progression.

### PI3K/Akt Pathway

In general, PI3K/Akt acts as a usual transduction molecule in fibrotic diseases, is not only related to promote the production of collagen, but also to participate in the activation of myofibroblasts (56). Recent experiments have been shown that phosphorylation level of Akt is increased in liver fibrosis, which is regarding as a signal of eNOS expression, and resulting in production of NO (57). On the contrary, the inhibition of Akt/eNOS is confirmed by liver GRK2 in STZ-induced diabetic mice (58). They suggest that the direct binding between GRK2 and Akt could disrupt the latter membrane attachment (59). Liu et al. (60) has found that eNOS activation is down-regulated in endothelial diseases *via* the interaction of GRK2 with Akt, ultimately reduced NO production. However, another research suggests that stabilizing GRK2 kinase contributes to activating PI3K/Akt signaling pathway and promoting the development of gallbladder cancer (61). Moreover, it has been observed that the activation of the PI3K/Akt by IGF-1 could reverse the degradation of GRK2, leading to the enhancement of its stability and the upregulation of kinase levels (62). Besides this, emerging evidence have indicated that T cell cytokine secretion is due to the activation of PI3Kγ signaling, the direct protein-protein interaction between PI3Kγ and GRK2, which is depended on the Ser-197 site of PI3K molecule. GRK2 activity is essential to the transactivation of CXCR4 and TCR–CXCR4 complex formation, deriving the activation of PI3Kγ signaling to promote the secretion of IL-2 and IL-10 (63). These findings confirm that there is a mutual crosstalk relationship between GRK2 and PI3K, which related to membrane targeting, kinase activity and levels of GRK2.

### ERK1/2 Pathway

It has been demonstrated that the proteasomal degradation of GRK2 could be interrupted through ERK phosphorylated it on Ser-670 (64). It is beneficial to enhance p-GRK2 level consists with p-ERK1/2 increased, which appears to enlarge myocardial infarction and fibrotic area, as well as collagen synthesis. When the ERK1/2 signaling is blocked in mice CFs, the expression of p-GRK2 is obviously reduced (65). Whereas other studies have demonstrated that the proliferation of CFs stimulated with arginine vasopressin (AVP) is obviously increased, which through V_1A_R-mediated GRK2/β-arrestin/ERK1/2 signaling (66). Simultaneously, another results suggest that silencing GRK2 could inhibit the continuous production of p-ERK1/2 induced by AVP then decreased caspase-3/7 activity and H9c2 cell survival (67, 68). Likewise, in A7r5 cells, inhibition of GRK2 can reduce AVP-mediated sustained phosphorylation of ERK1/2 and epidermal growth factor receptor activation, cell proliferation is also inhibited (69). Based on the previous studies, how GRK2 affect the phosphorylation of ERK1/2 in fibrotic disease is not fully understood.

### P38 MAPK Pathway

P38 MAPK, as a classical subtypes of MAPKs, engaging in cell differentiation, mitogenesis, and apoptosis, etc. To date, secretion of inflammatory cytokines *via* p38 MAPK activation leads to fibrosis emerged (liver, renal, lung and heart) (70, 71). Zhao et al. (72) reveals that aggravated p-p38 expression appears to promote the polarization of M1 macrophages, and induced monocyte infiltration which is contributed to liver fibrosis. Furthermore, Liu et al. (73) has determined that p38 induced GRK2 phosphorylation at Ser-670 contributes to enhancing LPS-stimulated monocyte migration. However, it is reported that GRK2 upregulates the phosphorylation of p38 to increase antigen-induced mast cell degranulation and cytokines production (74). On the contrary, GRK2, as a negative regulator, appears to phosphorylate p38 at Thr-123 contributes to inhibiting p38-mediated signaling transduction (75). Consequently, there is a crosstalk mechanism between GRK2 and p38, especially in fibrosis, which has not been claimed obviously.

### Nuclear Factor κB (NF-κB) Signaling Pathway

It is a well-known concept that NF-κB regulates a wide variety of biological responses and regulates inflammatory factor production, including interlukine-6 (IL-6), IL-1 and other cytokines. Both in hepatic and renal fibrosis, activation of NF-κB signaling pathway is involved in the activation of fibroblasts and accumulation of ECM (76, 77). Moreover, there is an obvious relationship between many other fibrotic diseases and the activation of NF-κB regulated by multiple molecules (78). Sonika et al. (79) suggests that GRK2 negatively regulates NF-κB1-p105-ERK pathway in primary macrophages stimulated by lipopolysaccharide. However, another study recognized that the activation of NF-κB is eliminated by inhibition of GRK2, diminishing the production of IL-6 in AVP-stimulated rat CFs (80). Similar results are confirmed that GRK2 is affecting the ability of NF-κB translocation to the nucleus in post-ischemic cells. It has been reported that the increased ability of NF-κB translocation to the nucleus contributes to tumor necrosis factor alpha (TNF-α) production and the activation of myofibroblasts (81). At present, the up-regulated translocation of NF-κB coursed by oxidative stress, resulting in GRK2 membranous translocation to increase the phosphorylation of dopamine D1 receptor (D1R) (82). According to the above research, there is a conflict effect of GRK2 in the activation of NF-κB pathway. Meanwhile, it’s necessary for us to require more studies to investigate the relationship between GRK2 and multiple signaling pathways in fibrotic diseases.

## GRK2 and Fibrotic Disease

**Figure 3:**
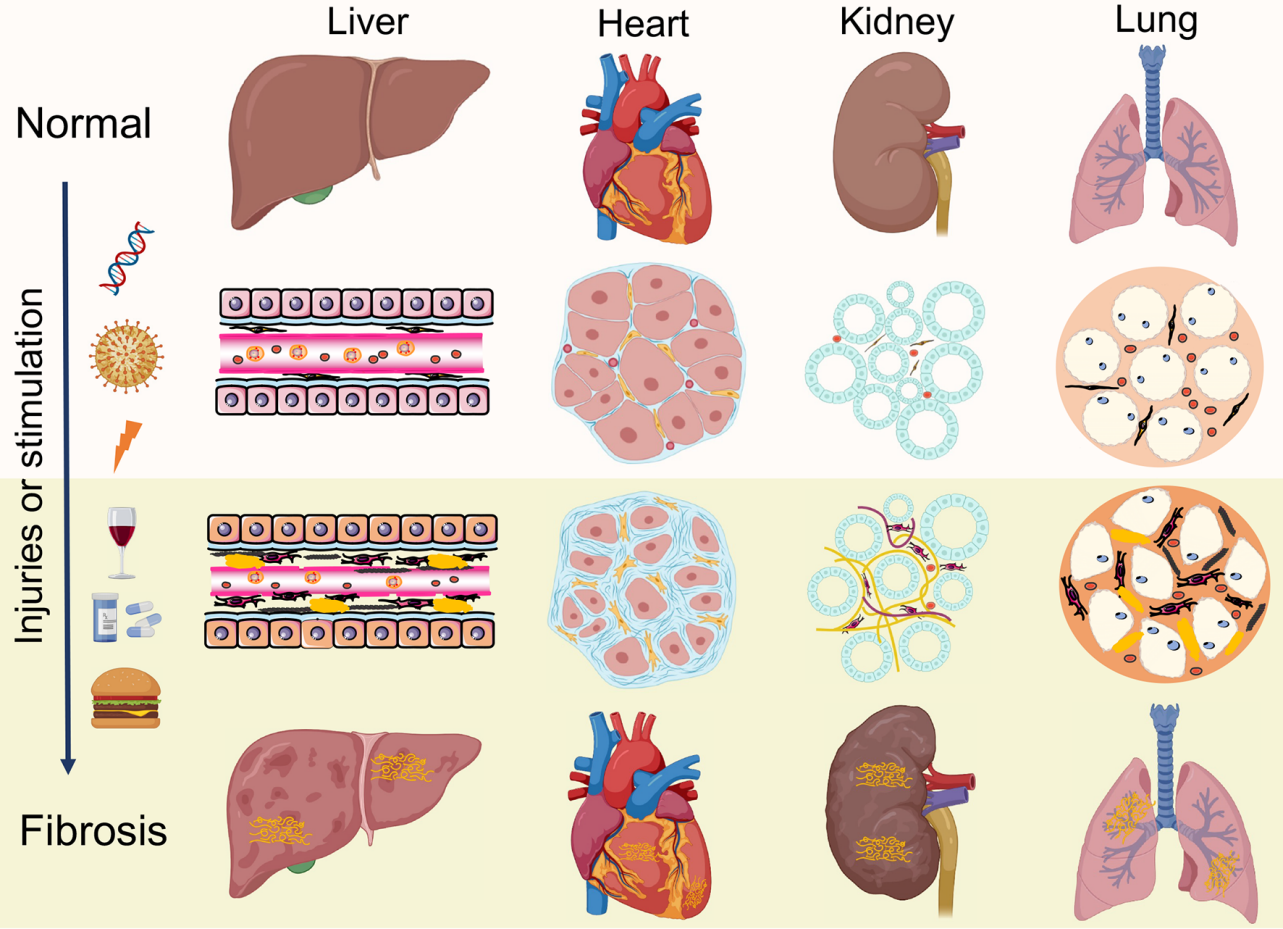
Similar pathological features of organ fibrosis. Fibrosis is a wound-healing process that leads to disruption of tissue architecture, organ dysfunction and eventually organ failure, which involves liver, heart, kidneys and lungs. Under continuous injuries or stimulation, the normal tissue repair response evolves into a diverse fibrotic process, including activation of fibroblasts, production of inflammatory cytokines and exaggerated accumulation of extracellular matrix (ECM).

**Figure 4:**
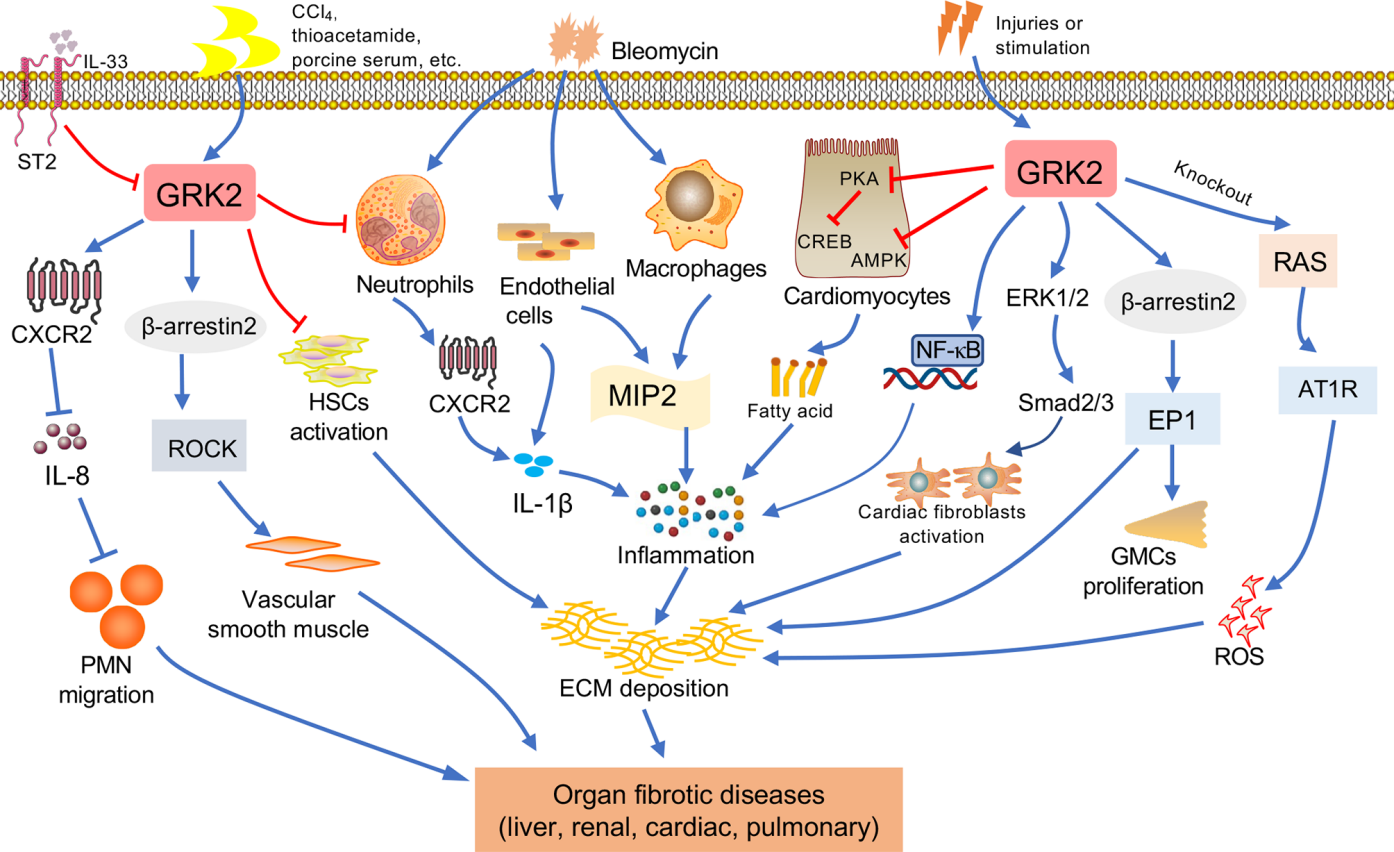
The relationship between GRK2 and fibrotic diseases. Various factors contribute to fibrosis progression in different tissues such as liver, kidney, lung, cardiac. When occurring the continuous pathological stimulus, the activated inflammatory cells and myofibroblasts will lead to extracellular matrix (ECM) production, resulting in fibrosis. Macrophages and endothelial cells can secrete cytokines. The immune cells including monocytes and neutrophils will secrete pro-inflammation factors, leading to the reactive oxygen species (ROS) and inflammation, and assist in the activation of myofibroblast. Important features of fibrous processes that are common in these organs include overproduction of cytokines, such as IL-1β, etc. Many injuries and stimulation will lead to the change of GRK2, GRK2 is involved in the pathological mechanism leading to the occurrence of organ fibrosis *via* multiple ways.

Under pathologic circumstances, the normal tissue repair response deviates from the homeostatic regulatory mechanisms and evolves into a diverse fibrotic process characterized by exaggerated deposition of ECM, which interrupts the normal organ architecture and ultimately becomes organ failure (83) (**Figure 3**). In general, fibroblasts are the principal resource of ECM. Additionally, many immune cells, such as macrophages, neutrophil, and thymus cells, participates in the development of organ fibrosis through secreting some pro-inflammatory or anti-inflammatory factors, including interferon-gamma (IFN-γ), matrix metalloproteinases (MMPs), IL-1β, IL-13, TNF-α, transforming growth factor-beta (TGF-β), etc (84) (**Figure 4**). At present, the treatment of fibrotic diseases is still a major difficulty. Accumulating evidence suggests the GRK2 signaling hub can influence different cells activation (85) and tissue fibrosis process, eventually leading to organic structure destruction and dysfunction (**Table 1**).

**Table 1:** Role of GRK2 in the development of multiple fibrotic diseases.

Type of fibrosis	Model	Relevant functional effects	Related mechanisms	References
**Cardiac fibrosis**	Isoproterenol-infused SHR	Cardiac hypertrophy; collagen I expression	GRK2-ADRB1	(86)
	Myocardial infarction mice	Collagen expression; cardiac dysfunction; heart failure	GRK2-βAR	(87)
	HFD mice	Cardiac myofibroblast; collagen deposition	GRK2-PKA-CREB; GRK2-AMPK	(48)
	Myocardial infarction model by thoracotomy, CF treated with PHPS1	Collagen I; collagen III deposition	GRK2-ERK1/2 -Smad2/3	(65)
	Neonatal rat CF treated with AVP	α-SMA; MMP2; MMP9; proliferation of CF	AVP-V_1A_R-GRK2 -β-arrestin-ERK1/2	(66)
	AVP-induced neonatal rat CF	Cardiac inflammation; fibroblast proliferation; cardiac remodeling	AVP-GRK2-NF-κB -IL-6	(80)
**Renal fibrosis**	shGRK2 mice	ROS; ECM; inhibition of matrix degradation	GRK2-RAS-AT1R	(88)
	HFD+STZ mice; GMCs induced by PGE_2_	ECM deposition; GMCs proliferation	GRK2-β-arrestin2 -EP1	(89)
	Chronic heart failure	Inflammation; ECM	GPCRs-Gβγ-GRK2	(90)
**Liver fibrosis**	Human patient tissue specimens	Recruitment of neutrophils in the liver	IL-33-ST2-GRK2 -CXCR2	(91)
	Cirrhotic rats induced by CCl_4_	The contractility of vascular smooth muscle	GRK2-β-arrestin2 -ROCK	(92)
	Porcine serum induced fibrotic rats	HSCs activation	Decreased expression of GRK2	(93)
	CCl_4_ or thioacetamide injected mice	HSCs activation; collagen deposition	GRK2-A_2A_R-MMPs	(94–96)
**Pulmonary fibrosis**	Bleomycin-induced pulmonary fibrotic mice	Recruitment of neutrophils; α-SMA; collagen-1; inflammation	GRK2-CXCR2-MIP2-IL-1β	(97)

SHR means the spontaneously hypertensive rats; PHPS1 used to specifically inhibit SHP-2; shGRK2 mice are the GRK2 hemizygous mice.

ADRB1, adrenergic receptor beta 1; HFD, high-fat diet; CREB, cAMP-response element binding protein; AVP, arginine vasopressin; CF, cardiac fibroblast; AMPK, AMP-activated protein kinase; MMP2, matrix metallopeptidase 2; RAS, renin-angiotensin system; STZ, streptozocin; GMCs, glomerular mesangial cells; HSCs, hepatic stellate cells; ROCK, Rho-kinase inhibitors; α-SMA, alpha-smooth muscle actin; ST2, serum stimulation-2.

### Liver Fibrosis

Liver fibrosis refers to the repeated destruction and regeneration of hepatocytes during the development of chronic liver disease, which leads to excessive deposition and abnormal distribution of ECM such as collagen, glycoproteins, and proteoglycans in the liver (98). This process also leads to the continuous activation of HSCs, which then leads to the formation of scar tissue in the liver and presents as abnormal wound healing. Activation of HSCs is considered to be a key step in the development of liver fibrosis (99). Upon stimulation of pro-fibrogenic factors, quiescent HSCs are activated to become myofibroblasts, which are characterized by enhanced proliferation and chemotaxis, and excessive ECM components production such as collagen I. Liver fibrosis may develop into cirrhosis, even ultimately leading to hepatocellular carcinoma (100). It’s urgent to find effective therapeutic targets for liver fibrosis because it contributes to increasing the incidence of morbidity in developed countries (101). In this regard, the researcher has reported that GRK2 is overexpressed in liver cirrhosis patients. Upregulation of GRK2 reduces migration activity of the polymorphonuclear neutrophils through activating IL-33/serum stimulation-2 (ST2 pathway), along with lower expression of membranous CXCR2 (91). Consistent with these findings, the increased expression of GRK2 has been found in cirrhotic patients and rats, accompanying with the aggravated interaction between the α1 adrenergic receptor and β-arrestin2 (92). Moreover, a porcine serum-induced liver fibrosis model in rats is used in our previous study, its pathological feature is more similar to humans in the pathogenesis of liver fibrosis. At the same time, we have found that the expression of GRK2 in liver tissue shows a downward trend with the extension of the modeling time, which is consistent with the expression tendency in rat HSCs. In addition, experiments also find lower GRK2 expression can promote the proliferation of HSCs and promote the occurrence of liver fibrosis (93). Adenosine is a purine nucleoside that acts on the adenosine A_2A_ receptor (A_2A_R), as well as participating in collagen production. Furthermore, depletion of A_2A_R would ameliorate the fibrosis (94). Further investigation has suggested that IFN-γ disrupts the expression of AC, which is required for A_2A_ to promote the production of collagen and exert anti-fibrotic effect. Meanwhile, TNF-α could enhance the activity of A_2A_R by impairing GRK2 and β-arrestin-mediated receptor desensitization. It suggested that GRK2 may also slow down the process of liver fibrosis by desensitizing A_2A_R in fibrotic tissue (95, 96). In the future research, we must pay more attention to potential role and mediated mechanisms of GRK2 in liver fibrosis.

### Myocardial Fibrosis

In the healthy heart, it seems essential to maintain both structure and integrity of the organ by ECM (102). Conversely, cardiac stress leads to excessive production of ECM, which ultimately causes the occurrence of cardiac fibrosis (103). Components of ECM including collagen I and III, laminin, fibronectin and matrix metalloproteinases, which are mainly produced by CFs (104). CFs, the main effector cells, in the course of cardiac fibrosis may proliferate, migrate, and differentiate into myofibroblasts, which is characterized by increased amount of alpha-smooth muscle actin (α-SMA) (105). Generally speaking, cardiac fibrosis has been linked to morbidity and mortality. Due to heart failure may be caused by cardiac fibrosis, which affecting 1%~2% of the world^’^s population live, it is a major public health concern (106, 107). Even though the reports have demonstrated that reversibility is definite in cardiac fibrotic, the mechanisms responsible for cardiac fibrosis is still unclear (108).

GRK2 has turned out to be crucial in cardiac contractility (87). The expression of GRK2 in myocardial depends on its level in peripheral blood mononuclear cells, likewise upregulation of GRK2 was shown in hypertrophy and hypertension (86). Currently, depletion of GRK2 in fibroblasts reduces the expression of fibrotic gene and diminished fibrosis in a post-ischemia-reperfusion (I/R) mice model (109). Otherwise, elevated level of AVP, which is secreted in response to hypovolemic of cardiac stress, has been considered as a potential risk factor of cardiac fibrosis. AVP induces the proliferation of CFs *via* GRK2/β-arrestin/ERK1/2 signaling pathway, while AVP-induced CFs proliferation is eliminated after GRK2 is blocked (66). Accordingly, other studies show that the up-regulated expression of p-GRK2 consisted with the activation of the ERK1/2/Smad2/3 signaling pathway is conducive to the production of collagen I/III in myocardial fibrosis mice (65). Moreover, GRK2-Ct peptide transfection (GRK2-Ct, inhibitory peptide of GRK2 activation) or GRK2 shRNA-mediated gene silencing abolishes AVP-evoked p-ERK1/2 and suppresses the expression of MMP2 and α-SMA in CFs, which could also inhibit the proliferation of CFs. The results of this study suggest that the low expression level of GRK2 could inhibit myocardial fibrosis. The degree of fibrosis is attenuated in the GRK2^flox/flox^ mice. However, knockdown of GRK2 significantly enhances carbachol-mediated activation of ERK1/2 *in vitro* (74). And researchers also have detected changes in GRK2 levels at different time points after I/R injury. Strikingly, they believe that the expression of GRK2 markedly diminished after myocardial I/R, Akt protein levels show a similar trend as GRK2 upon pre-conditioning. The data indicate that GRK2 phosphorylation at Ser-670 in ischemia or at Ser-685 in early reperfusion, which respectively aggravate proteasome- and calpain-mediated GRK2 degradation (110). Consistent with these findings, GRK2 is on a trajectory to become the most important target for cardiac fibrosis, and it is urgently to further explore the underlying mechanisms.

### Renal Fibrosis

During the past decades of years, the mortality of chronic kidney disease (CKD) has increased rapidly and continues to increase at an annual rate of 1% per year, which makes this life-threatening disease a global burden (111). Renal fibrosis, which is a common pathological result of most patients with advanced CKD (112). It is initially triggered by various biophysiological damages or inflammatory cytokines as a protective response to kidney damage. However, this reaction will become a pathogenic factor when kidney damage is prolonged and overreacted, eventually giving rise of end-stage kidney disease. Likewise, continuous renal inflammation, including secretion of pro-fibrotic factors, chemokines and cytokines, which could prompt the fibrosis (113). Renal fibrosis is a complicated disorder characterized by the destruction of kidney parenchyma, composed by the abnormal accumulation of ECM and lower glomerular filtration rate (114).

GRK2 plays a canonical role in regulating angiotensin II type 1 receptor (AT1R) desensitization, which is important in modulating a multitude of function in the kidney such as renal blood flow, glomerular filtration (115). GRK2 knockout rat model has been used to investigate the effects of GRK2 in kidney diseases, continues studies investigate that severer kidney-specific damage was occurred in GRK2 knockdown mice. Glomerular formation is altered during the development of GRK2 targeted knockout mice, which may be the basis for kidney shrinkage and functional decline in adult shGRK2 mice. In addition, the production of reactive oxygen species and ECM is increased in the GRK2 knockdown mice, especially the level of collagen rises obviously. The underlying mechanism possibly contributes to activating AT1R signaling through GRK2 depletion, instead of the expression of renal AT1R, which altering the renal function in shGRK2 mice (88). Whereas there is no obviously hypoplasia effect on other organs, it is worth noting that the regulation of GRK2-mediated AT1R signaling in nephrogenesis. There is another research presented that the increased expression of cytoplasm GRK2 may modulate the internalization of EP1 and promote the proliferation of glomerular mesangial cells, leading to the deposition of ECM (89, 116). Numerous preclinical studies confirmed that damaged kidney function, as an indicator of death in patients with ischemic and non-ischemic etiologies advanced chronic heart failure, have strongly linked to heart disease (117). Advance of CKD in the chronic phase of heart failure consists with altered GRK2 expression and membrane translocation *via* GPCRs-Gβγ, which promotes renal inflammation and fibrosis (90). Mechanistically, disrupting Gβγ-GRK2 signaling seems to play a major role in renal protection. As mentioned before, we need to be aware of the urgency of finding an effective strategy for anti-renal fibrosis, which is related to alteration of GRK2.

### Pulmonary Fibrosis

Pulmonary fibrosis caused by injury and infection is typically one of the chronic, prolonged pulmonary disease processes, along with excessive deposition of the ECM (118, 119). Moreover, multiple cytokines (including IFN-γ, TGF-β, IL-1β, TNF-α, IL-8 etc.) secreted by alveolar epithelial type cells, macrophages and other cells can promote the activation of fibroblasts, and also as an important risk for fibrosis (120). Roux et al. (121) has showed that IL-8, an important mediator of acute lung injury, could be elevated expression in rat and human alveolar epithelial type II cells. Furthermore, IL-8 inhibits β2AR agonist-stimulated alveolar epithelial fluid transport through the GRK2/PI3K signaling pathway. This effect appears to the aggravated acute lung injury, even resulting in pulmonary fibrosis and failure. Mak et al. (122) has used eight cases of normal lung tissues and nine lung tissues within by cystic fibrosis. The lung tissues are homogenized by centrifugation after grinding. In the cystic fibrosis tissues, there is a higher expression of GRK2 by Western blot. The level of GRK2 mRNA is detected by Northern blotting, and the same result is achieved. However, Chen et al. (97) suggests that nintedanib successfully down-regulates the expression of α-SMA, collagen-1, chemokine receptor 2 and very late antigen 4 in bleomycin-induced pulmonary fibrosis, as well as an upregulation of GRK2 activity in peripheral blood neutrophils. Previous effects lead to ameliorate the lung inflammation, fibrosis and neutrophil chemotaxis. Although GRK2 has been confirmed to be involved in the development of pulmonary fibrosis by regulating multiple signaling pathways, the exact role of GRK2 has not yet been clearly illustrated.

## Conclusion and Prospect

Accumulated evidence has focused on the essential role of GRK2 subtype in fibrotic diseases. With further exploration of research, more and more functions and potential value of GRK2 have been discovered. The expression of GRK2 could be used as biomarker to detect cardiac damage and determine the appropriate timing for clinical interventions even when this damage was not clinically very evident. Recently, plenty of fibrotic related pathways were shown to associate closely with the abnormal expression and activity of GRK2, and specific regulation in different cellular processes.

Fibrotic disease is one of the most difficult clinical problems in the world, thus blocking the further progress of fibrosis is crucial. Several methods have been designed to block GRK2 expression including congenital gene knockout. The application of GRK2 siRNA or GRK2 adenovirus can reduce the expression of GRK2 at the gene level. In addition, small molecule inhibitors and various peptides of GRK2 also have been applied to decrease the expression or activity of GRK2. Emerging strategies targeting GRK2 functionality are being investigated. Some methods have been identified to be effective in cell lines even in an animal model. The incidence of fibrosis is increasing globally, so it is necessary to understand the underlying mechanism of GRK2 in the process and take effective measures in time. During decades of research and exploration, GRK2 presents multiple functions in cardiac contractility, cell proliferation, inflammation and metabolic homeostasis. Simultaneously, several experiments suggested that the increased GRK2 in CFs induces the deposition of ECM, others also demonstrate the up-regulated GRK2 promotes the secretion of inflammatory cytokines in macrophages, then inducing fibroblasts activation (123). However, the decreased GRK2 is shown in the development of pulmonary fibrosis, and there is another result indicates that desensitization of A_2A_R by GRK2 may contribute to down-regulating the process of liver fibrosis. The data present that there is a difference of level of GRK2 in different fibrotic diseases, both expression and activity. GRK2 expression and activity were mainly detected in amounts of animal models rather than in enough clinical studies. Therefore, whether in animals or in clinic, it is more urgent to specifically explore how to target GRK2 in the treatments of fibrotic diseases. Meanwhile, it seems more indispensable to soft control GRK2 expression and function under pathological conditions to “normal” physiological states so as to restore the dynamic balance of cellular processes and prevent the development of organ fibrosis.

## Author Contributions

NL and SS wrote the manuscript. X-QL, T-TC, MQ, S-NZ, and Z-YW researched and organized the literature. L-LZ, WW, and W-YS contributed to manuscript revision. WW and W-YS are responsible for editing and funding acquisition. All authors contributed to the article and approved the submitted version.

## Funding

This work was supported by grants from the National Natural Science Foundation of China (No. 81770605), Research Level Improvement Program of Anhui Medical University (No.2021xkjT016), Improvement Program of Scientific Research Basement Construction (No.2021xkjT043), Program for Young Excellent Talents in Universities of Anhui Province (No. gxyqZD2018024).

## Conflict of Interest

The authors declare that the research was conducted in the absence of any commercial or financial relationships that could be construed as a potential conflict of interest.

## Publisher’s Note

All claims expressed in this article are solely those of the authors and do not necessarily represent those of their affiliated organizations, or those of the publisher, the editors and the reviewers. Any product that may be evaluated in this article, or claim that may be made by its manufacturer, is not guaranteed or endorsed by the publisher.

## References

[1] Penela P Murga C Ribas C Lafarga V Mayor F Jr. The Complex G Protein-Coupled Receptor Kinase 2 (GRK2) Interactome Unveils New Physiopathological Targets. Br J Pharmacol (2010) 160(4):821–32. doi: 10.1111/j.1476-5381.2010.00727.x PMC2935989 20590581

[2] Murga C Arcones AC Cruces-Sande M Briones AM Salaices M Mayor F Jr. G Protein-Coupled Receptor Kinase 2 (GRK2) as a Potential Therapeutic Target in Cardiovascular and Metabolic Diseases. Front Pharmacol (2019) 19:112(10). doi: 10.3389/fphar.2019.00112 PMC6390810 30837878

[3] Sun WY Wu JJ Peng WT Sun JC Wei W. The Role of G Protein-Coupled Receptor Kinases in the Pathology of Malignant Tumors. Acta Pharmacol Sin (2018) 39(11):1699–705. doi: 10.1038/s41401-018-0049-z PMC6289378 29921886

[4] Whalen EJ Foster MW Matsumoto A Ozawa K Violin JD Que LG. Regulation of β-Adrenergic Receptor Signaling by S-Nitrosylation of G-Protein-Coupled Receptor Kinase 2. Cell (2007) 129(3):511–22. doi: 10.1016/j.cell.2007.02.046 17482545

[5] Roehlen N Crouchet E Baumert TF. Liver Fibrosis: Mechanistic Concepts and Therapeutic Perspectives. Cells (2020) 9(4):875. doi: 10.3390/cells9040875 PMC7226751 32260126

[6] Zullo A Mancini FP Schleip R Wearing S Klingler W. Fibrosis: Sirtuins at the Checkpoints of Myofibroblast Differentiation and Profibrotic Activity. Wound Repair Regen (2021) 29(4):650–66. doi: 10.1111/wrr.12943 34077595

[7] Theret M Low M Rempel L Li FF Tung LW Contreras O. *In Vitro* Assessment of Anti-Fibrotic Drug Activity Does Not Predict *In Vivo* Efficacy in Murine Models of Duchenne Muscular Dystrophy. Life Sci (2021) 279:119482. doi: 10.1016/j.lfs.2021.119482 33891939

[8] Dolivo D Weathers P Dominko T. Artemisinin and Artemisinin Derivatives as Anti-Fibrotic Therapeutics. Acta Pharm Sin B (2021) 11(2):322–39. doi: 10.1016/j.apsb.2020.09.001 PMC7893118 33643815

[9] Shumar JN Chandel A King CS. Antifibrotic Therapies and Progressive Fibrosing Interstitial Lung Disease (PF-ILD): Building on INBUILD. J Clin Med (2021) 10(11):2285. doi: 10.3390/jcm10112285 34070297 PMC8197477

[10] Cannavo A Liccardo D Koch WJ. Targeting Cardiac β-Adrenergic Signaling *via* GRK2 Inhibition for Heart Failure Therapy. Front Physiol (2013) 4:264. doi: 10.3389/fphys.2013.00264 24133451 PMC3783981

[11] Gomes RN Manuel F Nascimento DS. The Bright Side of Fibroblasts: Molecular Signature and Regenerative Cues in Major Organs. NPJ Regener Med (2021) 6(1):43. doi: 10.1038/s41536-021-00153-z PMC8355260 34376677

[12] Weiskirchen R Weiskirchen S Tacke F. Organ and Tissue Fibrosis: Molecular Signals, Cellular Mechanisms and Translational Implications. Mol Aspects Med (2019) 65:2–15. doi: 10.1016/j.mam.2018.06.003 29958900

[13] Ribas C Penela P Murga C Salcedo A García-Hoz C Jurado-Pueyo M. The G Protein-Coupled Receptor Kinase (GRK) Interactome: Role of GRKs in GPCR Regulation and Signaling. Biochim Biophys Acta (2007) 1768(4):913–22. doi: 10.1016/j.bbamem.2006.09.019 17084806

[14] Laudette M Formoso K Lezoualc’h F. GRKs and Epac1 Interaction in Cardiac Remodeling and Heart Failure. Cells (2021) 10(1):154. doi: 10.3390/cells10010154 33466800 PMC7830799

[15] Nogués L Palacios-García J Reglero C Rivas V Neves M Ribas C. G Protein-Coupled Receptor Kinases (GRKs) in Tumorigenesis and Cancer Progression: GPCR Regulators and Signaling Hubs. Semin Cancer Biol (2018) 48:78–90. doi: 10.1016/j.semcancer.2017.04.013 28473253

[16] Penela P Ribas C Sánchez-Madrid F Mayor F Jr. G Protein-Coupled Receptor Kinase 2 (GRK2) as a Multifunctional Signaling Hub. Cell Mol Life Sci (2019) 76(22):4423–46. doi: 10.1007/s00018-019-03274-3 PMC6841920 31432234

[17] Penela P Murga C Ribas C Salcedo A Jurado-Pueyo M Rivas V. G Protein-Coupled Receptor Kinase 2 (GRK2) in Migration and Inflammation. Arch Physiol Biochem (2008) 114(3):195–200. doi: 10.1080/13813450802181039 18618354

[18] Penela P Elorza A Sarnago S Mayor F Jr. Beta-Arrestin- and C-Src-Dependent Degradation of G-Protein-Coupled Receptor Kinase 2. EMBO J (2001) 20(18):5129–38. doi: 10.1093/emboj/20.18.5129 PMC125273 11566877

[19] Wan KF Sambi BS Tate R Waters C Pyne NJ. The Inhibitory Gamma Subunit of the Type 6 Retinal cGMP Phosphodiesterase Functions to Link C-Src and G-Protein-Coupled Receptor Kinase 2 in a Signaling Unit That Regulates P42/P44 Mitogen-Activated Protein Kinase by Epidermal Growth Factor. J Biol Chem (2003) 278(20):18658–63. doi: 10.1074/jbc.M212103200 12624098

[20] Sallese M Mariggiò S D’Urbano E Iacovelli L De Blasi A. Selective Regulation of Gq Signaling by G Protein-Coupled Receptor Kinase 2: Direct Interaction of Kinase N Terminus With Activated Galphaq. Mol Pharmacol (2000) 57(4):826–31. doi: 10.1124/mol.57.4.826 10727532

[21] Chuang TT LeVine H 3rd De Blasi A. Phosphorylation and Activation of Beta-Adrenergic Receptor Kinase by Protein Kinase C. J Biol Chem (1995) 270(31):18660–5. doi: 10.1074/jbc.270.31.18660 7629197

[22] Mandyam CD Thakker DR Christensen JL Standifer KM. Orphanin FQ/nociceptin-Mediated Desensitization of Opioid Receptor-Like 1 Receptor and Mu Opioid Receptors Involves Protein Kinase C: A Molecular Mechanism for Heterologous Cross-Talk. J Pharmacol Exp Ther (2002) 302(2):502–9. doi: 10.1124/jpet.102.033159 12130708

[23] Pitcher JA Tesmer JJ Freeman JL Capel WD Stone WC Lefkowitz RJ. Feedback Inhibition of G Protein-Coupled Receptor Kinase 2 (GRK2) Activity by Extracellular Signal-Regulated Kinases. J Biol Chem (1999) 274(49):34531–4. doi: 10.1074/jbc.274.49.34531 10574913

[24] Taguchi K Sakata K Ohashi W Imaizumi T Imura J Hattori Y. Tonic Inhibition by G Protein-Coupled Receptor Kinase 2 of Akt/endothelial Nitric-Oxide Synthase Signaling in Human Vascular Endothelial Cells Under Conditions of Hyperglycemia With High Insulin Levels. J Pharmacol Exp Ther (2014) 349(2):199–208. doi: 10.1124/jpet.113.211854 24570070

[25] Murthy KS Mahavadi S Huang J Zhou H Sriwai W. Phosphorylation of GRK2 by PKA Augments GRK2-Mediated Phosphorylation, Internalization, and Desensitization of VPAC2 Receptors in Smooth Muscle. Am J Physiol Cell Physiol (2008) 294(2):C477–87. doi: 10.1152/ajpcell.00229.2007 18077607

[26] Cong M Perry SJ Lin FT Fraser ID Hu LA Chen W. Regulation of Membrane Targeting of the G Protein-Coupled Receptor Kinase 2 by Protein Kinase A and its Anchoring Protein AKAP79. J Biol Chem (2001) 276(18):15192–9. doi: 10.1074/jbc.M009130200 11278469

[27] Evron T Daigle TL Caron MG. GRK2: Multiple Roles Beyond G Protein-Coupled Receptor Desensitization. Trends Pharmacol Sci (2012) 33(3):154–64. doi: 10.1016/j.tips.2011.12.003 PMC3294176 22277298

[28] Lorenz K Lohse MJ Quitterer U. Protein Kinase C Switches the Raf Kinase Inhibitor From Raf-1 to GRK-2. Nature (2003) 426:574–9. doi: 10.1038/nature02158 14654844

[29] Gui YJ Liao CX Liu Q Guo Y Xu DY. RKIP Corrects Impaired Beta (2)-Adrenergic Receptor Vasodilatation in Hypertension by Downregulation of GRK2. Int J Cardiol (2016) 207:359–60. doi: 10.1016/j.ijcard.2016.01.191 26820366

[30] Carman CV Lisanti MP Benovic JL. Regulation of G Protein-Coupled Receptor Kinases by Caveolin. J Biol Chem (1999) 274(13):8858–64. doi: 10.1074/jbc.274.13.8858 10085129

[31] Rybin VO Xu X Lisanti MP Steinberg SF. Differential Targeting of Beta -Adrenergic Receptor Subtypes and Adenylyl Cyclase to Cardiomyocyte Caveolae. A Mechanism to Functionally Regulate the cAMP Signaling Pathway. J Biol Chem (2000) 275(52):41447–57. doi: 10.1074/jbc.M006951200 11006286

[32] Mangmool S Haga T Kobayashi H Kim KM Nakata H Nishida M. Clathrin Required for Phosphorylation and Internalization of Beta2-Adrenergic Receptor by G Protein-Coupled Receptor Kinase 2 (GRK2). J Biol Chem (2006) 281(42):31940–9. doi: 10.1074/jbc.M602832200 16920721

[33] Zhang X Zheng M Kim KM. GRK2-Mediated Receptor Phosphorylation and Mdm2-Mediated β-Arrestin2 Ubiquitination Drive Clathrin-Mediated Endocytosis of G Protein-Coupled Receptors. Biochem Biophys Res Commun (2020) 533(3):383–90. doi: 10.1016/j.bbrc.2020.09.030 32962859

[34] Kang JH Toita R Kawano T Murata M Asai D. Design of Substrates and Inhibitors of G Protein-Coupled Receptor Kinase 2 (GRK2) Based on its Phosphorylation Reaction. Amino Acids (2020) 52(6-7):863–70. doi: 10.1007/s00726-020-02864-x 32577910

[35] Bosakova M Abraham SP Nita A Hruba E Buchtova M Taylor SP. Mutations in GRK2 Cause Jeune Syndrome by Impairing Hedgehog and Canonical Wnt Signaling. EMBO Mol Med (2020) 12(11):e11739. doi: 10.15252/emmm.201911739 33200460 PMC7645380

[36] Cant SH Pitcher JA. G Protein-Coupled Receptor Kinase 2-Mediated Phosphorylation of Ezrin is Required for G Protein-Coupled Receptor-Dependent Reorganization of the Actin Cytoskeleton. Mol Biol Cell (2005) 16(7):3088–99. doi: 10.1091/mbc.e04-10-0877 PMC1165394 15843435

[37] Penela P Lafarga V Tapia O Rivas V Nogués L Lucas E. Roles of GRK2 in Cell Signaling Beyond GPCR Desensitization: GRK2-HDAC6 Interaction Modulates Cell Spreading and Motility. Sci Signal (2012) 5(224):pt3. doi: 10.1126/scisignal.2003098 22589388

[38] Crudden C Shibano T Song D Dragomir MP Cismas S Serly J. Inhibition of G Protein-Coupled Receptor Kinase 2 Promotes Unbiased Downregulation of IGF1 Receptor and Restrains Malignant Cell Growth. Cancer Res (2021) 81(2):501–14. doi: 10.1158/0008-5472.CAN-20-1662 33158816

[39] Lee K. Epac: New Emerging cAMP-Binding Protein. BMB Rep (2021) 54(3):149–56. doi: 10.5483/BMBRep.2021.54.3.233 PMC8016657 33298248

[40] Chen C Du J Feng W Song Y Lu Z Xu M. β-Adrenergic Receptors Stimulate Interleukin-6 Production Through Epac-Dependent Activation of Pkcδ/P38 MAPK Signalling in Neonatal Mouse Cardiac Fibroblasts. Br J Pharmacol (2012) 166(2):676–88. doi: 10.1111/j.1476-5381.2011.01785 PMC3417497 22103274

[41] Che X Wang X Zhang J Peng C Zhen Y Shao X. Vitexin Exerts Cardioprotective Effect on Chronic Myocardial Ischemia/Reperfusion Injury in Rats *via* Inhibiting Myocardial Apoptosis and Lipid Peroxidation. Am J Transl Res (2016) 8(8):3319–28. PMC5009384 27648122

[42] Eijkelkamp N Wang H Garza-Carbajal A Willemen HL Zwartkruis FJ Wood JN. Low Nociceptor GRK2 Prolongs Prostaglandin E2 Hyperalgesia *via* Biased cAMP Signaling to Epac/Rap1, Protein Kinase C, and MEK/ERK. J Neurosci (2010) 30(38):12806–15. doi: 10.1523/jneurosci.3142-10.2010 PMC6633564 20861385

[43] Singhmar P Huo X Eijkelkamp N Berciano SR Baameur F Mei FC. Critical Role for Epac1 in Inflammatory Pain Controlled by GRK2-Mediated Phosphorylation of Epac1. Proc Natl Acad Sci U S A (2016) 113(11):3036–41. doi: 10.1073/pnas.1516036113 PMC4801297 26929333

[44] Almahariq M Mei FC Cheng X. Cyclic AMP Sensor EPAC Proteins and Energy Homeostasis. Trends Endocrinol Metab (2014) 25(2):60–71. doi: 10.1016/j.tem.2013.10.004 24231725 PMC3946731

[45] Yang Y Wang H Lv X Wang Q Zhao H Yang F. Involvement of cAMP-PKA Pathway in Adenosine A1 and A2A Receptor-Mediated Regulation of Acetaldehyde-Induced Activation of HSCs. Biochimie (2015) 115:59–70. doi: 10.1016/j.biochi.2015.04.019 25956975

[46] Han CC Liu Q Zhang Y Li YF Cui DQ Luo TT. CP-25 Inhibits PGE2-Induced Angiogenesis by Down-Regulating EP4/AC/cAMP/PKA-Mediated GRK2 Translocation. Clin Sci (Lond) (2020) 134(3):331–47. doi: 10.1042/CS20191032 31967309

[47] Huang J Mahavadi S Sriwai W Grider JR Murthy KS. Cross-Regulation of VPAC(2) Receptor Desensitization by M(3) Receptors via PKC-Mediated Phosphorylation of RKIP and Inhibition of GRK2. Am J Physiol Gastrointest Liver Physiol (2007) 292(3):G867–74. doi: 10.1152/ajpgi.00326.2006 17170028

[48] Tanaka S Imaeda A Matsumoto K Maeda M Obana M Fujio Y. β2-Adrenergic Stimulation Induces Interleukin-6 by Increasing Arid5a, a Stabilizer of mRNA, Through cAMP/PKA/CREB Pathway in Cardiac Fibroblasts. Pharmacol Res Perspect (2020) 8(2):e00590. doi: 10.1002/prp2.590 32302067 PMC7164407

[49] Morris R Kershaw NJ Babon JJ. The Molecular Details of Cytokine Signaling *via* the JAK/STAT Pathway. Protein Sci (2018) 27(12):1984–2009. doi: 10.1002/pro.3519 30267440 PMC6237706

[50] Papaioannou I Xu S Denton CP Abraham DJ Ponticos M. STAT3 Controls COL1A2 Enhancer Activation Cooperatively With JunB, Regulates Type I Collagen Synthesis Posttranscriptionally, and is Essential for Lung Myofibroblast Differentiation. Mol Biol Cell (2018) 29(2):84–95. doi: 10.1091/mbc.E17-06-0342 29142074 PMC5909935

[51] Hui J Gao J Wang Y Zhang J Han Y Wei L. Panax Notoginseng Saponins Ameliorates Experimental Hepatic Fibrosis and Hepatic Stellate Cell Proliferation by Inhibiting the Jak2/ Stat3 Pathways. J Tradit Chin Med (2016) 36(2):217–24. doi: 10.1016/s0254-6272(16)30030-9 27400477

[52] Eid RA Alkhateeb MA El-Kott AF Eleawa SM Zaki MSA Alaboodi SA. A High-Fat Diet Rich in Corn Oil Induces Cardiac Fibrosis in Rats by Activating JAK2/STAT3 and Subsequent Activation of ANG II/TGF-1β/Smad3 Pathway: The Role of ROS and IL-6 Trans-Signaling. J Food Biochem (2019) 43(8):e12952. doi: 10.1111/jfbc.12952 31368573

[53] Palikhe S Ohashi W Sakamoto T Hattori K Kawakami M Andoh T. Regulatory Role of GRK2 in the TLR Signaling-Mediated iNOS Induction Pathway in Microglial Cells. Front Pharmacol (2019) 10:59. doi: 10.3389/fphar.2019.00059 30778300 PMC6369205

[54] Iwakiri Y. Nitric Oxide in Liver Fibrosis: The Role of Inducible Nitric Oxide Synthase. Clin Mol Hepatol (2015) 21(4):319–25. doi: 10.3350/cmh.2015.21.4.319 PMC4712158 26770919

[55] Kawakami M Hattori M Ohashi W Fujimori T Hattori K Takebe M. Role of G Protein-Coupled Receptor Kinase 2 in Oxidative and Nitrosative Stress-Related Neurohistopathological Changes in a Mouse Model of Sepsis-Associated Encephalopathy. J Neurochem (2018) 145:474–88. doi: 10.1111/jnc.14329 29500815

[56] Higgins DF Ewart LM Masterson E Tennant S Grebnev G Prunotto M. BMP7-Induced-Pten Inhibits Akt and Prevents Renal Fibrosis. Biochim Biophys Acta Mol Basis Dis (2017) 1863(12):3095–104. doi: 10.1016/j.bbadis.2017.09.011 28923783

[57] Zheng Y Wang J Zhao T Wang L Wang J. Modulation of the VEGF/AKT/eNOS Signaling Pathway to Regulate Liver Angiogenesis to Explore the Anti-Hepatic Fibrosis Mechanism of Curcumol. J Ethnopharmacol (2021) 280:114480. doi: 10.1016/j.jep.2021.114480 34358654

[58] Taguchi K Hida M Hasegawa M Narimatsu H Matsumoto T Kobayashi T. Suppression of GRK2 Expression Reduces Endothelial Dysfunction by Restoring Glucose Homeostasis. Sci Rep (2017) 7(1):8436. doi: 10.1038/s41598-017-08998-5 28814745 PMC5559446

[59] Taguchi K Kobayashi T Takenouchi Y Matsumoto T Kamata K. Angiotensin II Causes Endothelial Dysfunction *via* the GRK2/Akt/eNOS Pathway in Aortas From a Murine Type 2 Diabetic Model. Pharmacol Res (2011) 64(5):535–46. doi: 10.1016/j.phrs.2011.05.001 21571071

[60] Liu S Premont RT Kontos CD Zhu S Rockey DC. A Crucial Role for GRK2 in Regulation of Endothelial Cell Nitric Oxide Synthase Function in Portal Hypertension. Nat Med (2005) 11(9):952–8. doi: 10.1038/nm1289 16142243

[61] Zhang F Xiang S Cao Y Li M Ma Q Liang H. EIF3D Promotes Gallbladder Cancer Development by Stabilizing GRK2 Kinase and Activating PI3K-AKT Signaling Pathway. Cell Death Dis (2017) 8(6):e2868. doi: 10.1038/cddis.2017.263 28594409 PMC5520919

[62] Salcedo A Mayor F Jr Penela P. Mdm2 is Involved in the Ubiquitination and Degradation of G-Protein-Coupled Receptor Kinase 2. EMBO J (2006) 25(20):4752–62. doi: 10.1038/sj.emboj.7601351 PMC1618114 17006543

[63] Dinkel BA Kremer KN Rollins MR Medlyn MJ Hedin KE. GRK2 Mediates TCR-Induced Transactivation of CXCR4 and TCR-CXCR4 Complex Formation That Drives PI3Kγ/PREX1 Signaling and T Cell Cytokine Secretion. J Biol Chem (2018) 293(36):14022–39. doi: 10.1074/jbc.RA118.003097 PMC6130939 30018141

[64] Elorza A Penela P Sarnago S Mayor F Jr. MAPK-Dependent Degradation of G Protein-Coupled Receptor Kinase 2. J Biol Chem (2003) 278(31):29164–73. doi: 10.1074/jbc.M304314200 12738776

[65] Lu YG Tan H Ma Q Li XX Cui J Zhang X. SH2 Domain-Containing Protein Tyrosine Phosphatase-2 (SHP-2) Prevents Cardiac Remodeling After Myocardial Infarction Through ERK/SMAD Signaling Pathway. Hum Cell (2021) 34(2):325–34. doi: 10.1007/s13577-020-00430-x 33415691

[66] Chen Y Xu F Zhang L Wang X Wang Y Woo AY. Grk2/β-Arrestin Mediates Arginine Vasopressin-Induced Cardiac Fibroblast Proliferation. Clin Exp Pharmacol Physiol (2017) 44(2):285–93. doi: 10.1111/1440-1681.12696 27862165

[67] Wu P Ye S Li M Li H Kan Y Yang Z. Impurity Identification and Quantification for Arginine Vasopressin by Liquid Chromatography/High-Resolution Mass Spectrometry. Rapid Commun Mass Spectrom (2020) 34(12):e8799. doi: 10.1002/rcm.8799 32247289

[68] Zhu W Tilley DG Myers VD Coleman RC Feldman AM. Arginine Vasopressin Enhances Cell Survival *via* a G Protein–Coupled Receptor Kinase 2/β-Arrestin1/Extracellular-Regulated Kinase 1/2-Dependent Pathway in H9c2 Cells. Mol Pharmacol (2013) 84(2):227–35. doi: 10.1124/mol.113.086322 PMC3716325 23690069

[69] Zhang L Wang X Cao H Chen Y Chen X Zhao X. Vasopressin V_1A_ Receptor Mediates Cell Proliferation Through GRK2-EGFR-ERK1/2 Pathway in A7r5 Cells. Eur J Pharmacol (2016) 792:15–25. doi: 10.1016/j.ejphar.2016.10.023 27773680

[70] Liu N Feng J Lu X Yao Z Liu Q Lv Y. Isorhamnetin Inhibits Liver Fibrosis by Reducing Autophagy and Inhibiting Extracellular Matrix Formation *via* the TGF-β1/Smad3 and TGF-β1/P38 MAPK Pathways. Mediators Inflamm (2019) 2019:6175091. doi: 10.1155/2019/6175091 31467486 PMC6701280

[71] Wei YQ Guo YF Yang SM Ma HH Li J. MiR-340-5p Mitigates the Proliferation and Activation of Fibroblast in Lung Fibrosis by Targeting TGF-β/P38/ATF1 Signaling Pathway. Eur Rev Med Pharmacol Sci (2020) 24(11):6252–61. doi: 10.26355/eurrev_202006_21523 32572892

[72] Zhao XA Chen G Liu Y Chen Y Wu H Xiong Y. Curcumin Reduces Ly6Chi Monocyte Infiltration to Protect Against Liver Fibrosis by Inhibiting Kupffer Cells Activation to Reduce Chemokines Secretion. BioMed Pharmacother (2018) 106:868–78. doi: 10.1016/j.biopha.2018.07.028 30119257

[73] Liu Z Jiang Y Li Y Wang J Fan L Scott MJ. TLR4 Signaling Augments Monocyte Chemotaxis by Regulating G Protein-Coupled Receptor Kinase 2 Translocation. J Immunol (2013) 191(2):857–64. doi: 10.4049/jimmunol.1300790 PMC3702632 23772028

[74] Subramanian H Gupta K Parameswaran N Ali H. Regulation of Fc∈RI Signaling in Mast Cells by G Protein-Coupled Receptor Kinase 2 and its RH Domain. J Biol Chem (2014) 289(30):20917–27. doi: 10.1074/jbc.M113.523969 PMC4110298 24904059

[75] Peregrin S Jurado-Pueyo M Campos PM Sanz-Moreno V Ruiz-Gomez A Crespo P. Phosphorylation of P38 by GRK2 at the Docking Groove Unveils a Novel Mechanism for Inactivating P38mapk. Curr Biol (2006) 16(20):2042–7. doi: 10.1016/j.cub.2006.08.083 17055984

[76] Zhang T Hu J Wang X Zhao X Li Z Niu J. MicroRNA-378 Promotes Hepatic Inflammation and Fibrosis *via* Modulation of the NF-κb-Tnfα Pathway. J Hepatol (2019) 70(1):87–96. doi: 10.1016/j.jhep.2018.08.026 30218679 PMC6554744

[77] Zhang L Liu L Bai M Liu M Wei L Yang Z. Hypoxia-Induced HE4 in Tubular Epithelial Cells Promotes Extracellular Matrix Accumulation and Renal Fibrosis via NF-κB. FASEB J (2020) 34(2):2554–67. doi: 10.1096/fj.201901950R 31909536

[78] Cartwright T Perkins ND L Wilson C. NFKB1: A Suppressor of Inflammation, Ageing and Cancer. FEBS J (2016) 283(10):1812–22. doi: 10.1111/febs.13627 26663363

[79] Patial S Saini Y Parvataneni S Appledorn DM Dorn GW 2nd Lapres JJ. Myeloid-Specific GPCR Kinase-2 Negatively Regulates NF-κb1p105-ERK Pathway and Limits Endotoxemic Shock in Mice. J Cell Physiol (2011) 226(3):627–37. doi: 10.1002/jcp.22384 PMC3013243 20717897

[80] Xu F Sun S Wang X Ni E Zhao L Zhu W. GRK2 Mediates Arginine Vasopressin-Induced Interleukin-6 Production *via* Nuclear Factor-κb Signaling Neonatal Rat Cardiac Fibroblast. Mol Pharmacol (2017) 92(3):278–84. doi: 10.1124/mol.116.107698 28193640

[81] Zhang QH Hao JW Li GL Ji XJ Yao XD Dong N. Proinflammatory Switch From Gαs to Gαi Signaling by Glucagon-Like Peptide-1 Receptor in Murine Splenic Monocyte Following Burn Injury. Inflamm Res (2018) 67:157–68. doi: 10.1007/s00011-017-1104-9 29022064

[82] Banday AA Fazili FR Lokhandwala MF. Oxidative Stress Causes Renal Dopamine D1 Receptor Dysfunction and Hypertension *via* Mechanisms That Involve Nuclear factor-kappaB and Protein Kinase C. J Am Soc Nephrol (2007) 18(5):1446–57. doi: 10.1681/ASN.2006121373 17409305

[83] Henderson NC Rieder F Wynn TA. Fibrosis: From Mechanisms to Medicines. Nature (2020) 587(7835):555–66. doi: 10.1038/s41586-020-2938-9 PMC8034822 33239795

[84] Tacke F Zimmermann HW. Macrophage Heterogeneity in Liver Injury and Fibrosis. J Hepatol (2014) 60(5):1090–6. doi: 10.1016/j.jhep.2013.12.025 24412603

[85] Cheng J Klei LR Hubel NE Zhang M Schairer R Maurer LM. GRK2 Suppresses Lymphomagenesis by Inhibiting the MALT1 Proto-Oncoprotein. J Clin Invest. (2020) 130(2):1036–51. doi: 10.1172/JCI97040 PMC6994119 31961340

[86] Sun X Zhou M Wen G Huang Y Wu J Peng L. Paroxetine Attenuates Cardiac Hypertrophy *via* Blocking GRK2 and ADRB1 Interaction in Hypertension. J Am Heart Assoc (2021) 10(1):e016364. doi: 10.1161/JAHA.120.016364 33372534 PMC7955481

[87] Schumacher SM Gao E Zhu W Chen X Chuprun JK Feldman AM. Paroxetine-Mediated GRK2 Inhibition Reverses Cardiac Dysfunction and Remodeling After Myocardial Infarction. Sci Transl Med (2015) 7(277):277ra31. doi: 10.1126/scitranslmed.aaa0154 PMC4768806 25739765

[88] Tutunea-Fatan E Abd-Elrahman KS Thibodeau JF Holterman CE Holleran BJ Leduc R. GRK2 Knockdown in Mice Exacerbates Kidney Injury and Alters Renal Mechanisms of Blood Pressure Regulation. Sci Rep (2018) 8(1):11415. doi: 10.1038/s41598-018-29876-8 30061705 PMC6065385

[89] Ni WJ Tang LQ Zhou H Ding HH Qiu YY. Reno Protective Effect of Berberine *via* Regulating the PGE2 -EP1-Gαq-Ca(2+) Signalling Pathway in Glomerular Mesangial Cells of Diabetic Rats. J Cell Mol Med (2016) 20(8):1491–502. doi: 10.1111/jcmm.12837 PMC4956950 27098986

[90] Rudomanova V Blaxall BC. Targeting GPCR-Gβγ-GRK2 Signaling as a Novel Strategy for Treating Cardiorenal Pathologies. Biochim Biophys Acta Mol Basis Dis (2017) 1863(8):1883–92. doi: 10.1016/j.bbadis.2017.01.020 PMC5466897 28130200

[91] Artru F Bou Saleh M Maggiotto F Lassailly G Ningarhari M Demaret J. IL-33/ST2 Pathway Regulates Neutrophil Migration and Predicts Outcome in Patients With Severe Alcoholic Hepatitis. J Hepatol (2020) 72(6):1052–61. doi: 10.1016/j.jhep.2019.12.017 31953139

[92] Chen W Sang JY Liu DJ Qin J Huo YM Xu J. Desensitization of G-Protein-Coupled Receptors Induces Vascular Hypocontractility in Response to Norepinephrine in the Mesenteric Arteries of Cirrhotic Patients and Rats. Hepatobiliary Pancreat Dis Int (2013) 12(3):295–304. doi: 10.1016/s1499-3872(13)60047-8 23742775

[93] Li R Sun WY Wang L Wei W. The Expression of GRK2 in the Rats With Immunological Hepatic Fibrosis. Acta Universitatis Medicinalis Anhui (2010) 45(04):458–62. doi: 10.19405/j.cnki.issn1000-1492.2010.04.005

[94] Peng Z Borea PA Varani K Wilder T Yee H Chiriboga L. Adenosine Signaling Contributes to Ethanolinduced Fatty Liver in Mice. J Clin Invest (2009) 119:582–94. doi: 10.1172/jci37409 PMC2648683 19221436

[95] Khoa ND Postow M Danielsson J Cronstein BN. Tumor Necrosis Factor-Alpha Prevents Desensitization of Galphas-Coupled Receptors by Regulating GRK2 Association With the Plasma Membrane. Mol Pharmacol (2006) 69(4):1311–9. doi: 10.1124/mol.105.016857 16385076

[96] Olah ME Caldwell CC. Adenosine Receptors and Mammalian Toll-Like Receptors: Synergism in Macrophages. Mol Interv (2003) 3(7):370–4. doi: 10.1124/mi.3.7.370 14993458

[97] Chen WC Chen NJ Chen HP Yu WK Su VY Chen H. Nintedanib Reduces Neutrophil Chemotaxis *via* Activating GRK2 in Bleomycin-Induced Pulmonary Fibrosis. Int J Mol Sci (2020) 21(13):4735. doi: 10.3390/ijms21134735 PMC7370174 32630825

[98] Bao YL Wang L Pan HT Zhang TR Chen YH Xu SJ. Animal and Organoid Models of Liver Fibrosis. Front Physiol (2021) 26:66613(12). doi: 10.3389/fphys.2021.66613 PMC8187919 34122138

[99] Trautwein C Friedman SL Schuppan D Pinzani M. Hepatic Fibrosis: Concept to Treatment. J Hepatol (2015) 62:S15–24. doi: 10.1016/j.jhep.2015.02.039 25920084

[100] Hernandez-Gea V Friedman SL. Pathogenesis of Liver Fibrosis. Annu Rev Pathol (2011) 6:425–56. doi: 10.1146/annurev-pathol-011110-130246 21073339

[101] Nishimichi N Tsujino K Kanno K Sentani K Kobayashi T Chayama K. Induced Hepatic Stellate Cell Integrin, α8β1, Enhances Cellular Contractility and Tgfβ Activity in Liver Fibrosis. J Pathol (2021) 253(4):366–73. doi: 10.1002/path.5618 PMC7986747 33433924

[102] Abdelaziz Mohamed I Gadeau AP Hasan A Abdulrahman N Mraiche F. Osteopontin: A Promising Therapeutic Target in Cardiac Fibrosis. Cells (2019) 8(12):1558. doi: 10.3390/cells8121558 PMC6952988 31816901

[103] Schimmel K Jung M Foinquinos A José GS Beaumont J Bock K. Natural Compound Library Screening Identifies New Molecules for the Treatment of Cardiac Fibrosis and Diastolic Dysfunction. Circulation (2020) 141(9):751–67. doi: 10.1161/circulationaha.119.042559 PMC7050799 31948273

[104] Lai S Fu X Yang S Zhang S Lin Q Zhang M. G Protein-Coupled Receptor Kinase-2: A Potential Biomarker for Early Diabetic Cardiomyopathy. J Diabetes (2019) 12(3):247–58. doi: 10.1111/1753-0407.12991 PMC7064927 31680450

[105] Gao L Wang LY Liu ZQ Jiang D Wu SY Guo YQ. TNAP Inhibition Attenuates Cardiac Fibrosis Induced by Myocardial Infarction Through Deactivating TGF-β1/Smads and Activating P53 Signaling Pathways. Cell Death Dis (2020) 11(1):44. doi: 10.1038/s41419-020-2243-4 31969558 PMC6976710

[106] Travers JG Kamal FA Robbins J Yutzey KE Blaxall BC. Cardiac Fibrosis: The Fibroblast Awakens. Circ Res (2016) 118(6):1021–40. doi: 10.1161/circresaha.115.306565 PMC4800485 26987915

[107] Dang S Zhang Z Li K Zheng J Qian LL Liu XY. Blockade of β-Adrenergic Signaling Suppresses Inflammasome and Alleviates Cardiac Fibrosis. Ann Transl Med (2020) 8(4):127. doi: 10.21037/atm.2020.02.31 32175420 PMC7048978

[108] Zou LX Chen C Yan X Lin QY Fang J Li PB. Resveratrol Attenuates Pressure Overload-Induced Cardiac Fibrosis and Diastolic Dysfunction *via* PTEN/Akt/Smad2/3 and NF-κb Signaling Pathways. Mol Nutr Food Res (2019) 63(24):1900418. doi: 10.1002/mnfr.201900418 31655498

[109] Woodall MC Woodall BP Gao E Yuan A Koch WJ. Cardiac Fibroblast GRK2 Deletion Enhances Contractility and Remodeling Following Ischemia/Reperfusion Injury. Circ Res (2016) 119(10):1116–27. doi: 10.1161/circresaha.116.309538 PMC5085864 27601479

[110] Penela P Inserte J Ramos P Rodriguez S Garcia D Mayor F Jr. Degradation of GRK2 and Akt is an Early and Detrimental Event in Myocardial Ischemia/Reperfusion. EBioMedicine (2019) 48:605–18. doi: 10.1016/j.ebiom.2019.09.019 PMC6838402 31594751

[111] Gu Y Liu X Huang X Yu XQ Lan HY. TGF-β in Renal Fibrosis: Triumphs and Challenges. Future Med Chem (2020) 12(9):853–66. doi: 10.4155/fmc-2020-0005 32233802

[112] Yang K Li W Bai T Xiao Y Yu W Luo P. Mindin Deficiency Alleviates Renal Fibrosis Through Inhibiting NF-κb and TGF-β/Smad Pathways. J Cell Mol Med (2020) 24(10):5740–50. doi: 10.1111/jcmm.15236 PMC7214143 32253812

[113] Gu YY Dou JY Huang XR Liu XS Lan HY. Transforming Growth Factor-β and Long non-Coding RNA in Renal Inflammation and Fibrosis. Front Physiol (2021) 13:684236. doi: 10.3389/fphys.2021.684236 PMC8155637 34054586

[114] Wu F Zhao Y Shao Q Fang K Dong R Jiang S. Ameliorative Effects of Osthole on Experimental Renal Fibrosis *In Vivo* and *In Vitro* by Inhibiting IL-11/ERK1/2 Signaling. Front Pharmacol (2021) 13:646331. doi: 10.3389/fphar.2021.646331 PMC8155534 34054526

[115] Pollard CM Ghandour J Cora N Perez A Parker BM Desimine VL. GRK2-Mediated Crosstalk Between β-Adrenergic and Angiotensin II Receptors Enhances Adrenocortical Aldosterone Production *In Vitro* and *In Vivo* . Int J Mol Sci (2020) 21(2):574. doi: 10.3390/ijms21020574 PMC7013621 31963151

[116] Wolf G Butzmann U Wenzel UO. The Renin-Angiotensin System and Progression of Renal Disease: From Hemodynamics to Cell Biology. Nephron Physiol (2003) 93(1):P3–13. doi: 10.1159/000066656 12411725

[117] Kamal FA Travers JG Schafer AE Harari S Martinez FJ Olschewski H. G Protein-Coupled Receptor-G-Protein βγ-Subunit Signaling Mediates Renal Dysfunction and Fibrosis in Heart Failure. J Am Soc Nephrol (2016) 28(1):197–208. doi: 10.1681/asn.2015080852 27297948 PMC5198268

[118] Karampitsakos T Tzouvelekis A Chrysikos S Harari S Martinez FJ Olschewski H. Pulmonary Hypertension in Patients With Interstitial Lung Disease. Pulm Pharmacol Ther (2018) 50:38–46. doi: 10.1016/j.pupt.2018.03.002 29605286

[119] Li H Zhao C Li Z Yao K Zhang J Si W. Identification of Potential Pathogenic Super-Enhancers-Driven Genes in Pulmonary Fibrosis. Front Genet (2021) 12:644143. doi: 10.3389/fgene.2021.644143 34054916 PMC8153712

[120] Kolahian S Fernandez IE Eickelberg O Hartl D. Immune Mechanisms in Pulmonary Fibrosis. Am J Respir Cell Mol Biol (2016) 55(3):309–22. doi: 10.1165/rcmb.2016-0121TR 27149613

[121] Roux J McNicholas CM Carles M Goolaerts A Houseman BT Dickinson DA. IL-8 Inhibits cAMP-Stimulated Alveolar Epithelial Fluid Transport *via* a GRK2/PI3K-Dependent Mechanism. FASEB J (2013) 27(3):1095–106. doi: 10.1096/fj.12-219295 PMC3574281 23221335

[122] Mak JC Chuang TT Harris CA Barnes PJ. Increased Expression of G Protein-Coupled Receptor Kinases in Cystic Fibrosis Lung. Eur J Pharmacol (2002) 436(3):165–72. doi: 10.1016/s0014-2999(01)01625-9 11858796

[123] Yang XZ Wei W. CP-25, a Compound Derived From Paeoniflorin: Research Advance on Its Pharmacological Actions and Mechanisms in the Treatment of Inflammation and Immune Diseases. Acta Pharmacol Sin (2020) 41(11):1387–94. doi: 10.1038/s41401-020-00510-6 PMC7656585 32884075

